# Ultraviolet radiation: a double-edged sword in old forest *Lobaria* lichens—reducing growth while enhancing acclimation

**DOI:** 10.1007/s00442-025-05765-6

**Published:** 2025-07-18

**Authors:** Ida Karina Kann, Knut Asbjørn Solhaug, Yngvar Gauslaa

**Affiliations:** Faculty of Environmental Sciences and Natural Resource Management, Norwegian University of Life Sciences, P.O. Box 5003, NO-1432 Ås, Norway

**Keywords:** Light, *Lobaria pulmonaria*, Melanic pigmentation, Photoinhibition, Photosynthesis

## Abstract

This study examines the effects of three light regimes—1) photosynthetic light (PAR) only, 2) PAR + UV-A, and 3) PAR + UV-A + UV-B radiation—on area and biomass growth in two old forest lichens: the widely distributed *Lobaria pulmonaria*, noted for rapid growth, and the rarer *L. virens*, with previously undocumented growth rates but known susceptibility to high light. To overcome the constraints of artificial light sources, we utilized solar filters in sun-exposed field conditions to assess how UV impacts growth and influences mycobiont traits (melanic pigmentation, thallus thickness) and photobiont responses (Chl content, Chl fluorescence, CO_2_ uptake). While UV exposure significantly reduced growth rates, it did not impact photobiont processes. *Lobaria pulmonaria* demonstrated robust melanin synthesis under UV-B, preventing photoinhibition and safeguarding chlorophylls, whereas *L. virens*, with lower melanin production, showed reduced Chl content and increased vulnerability to solar radiation. Enhanced specific thallus mass, a proxy of water holding capacity, correlated with increased melanic pigmentation, suggesting that UV-B also aids in acclimation of drought tolerance. Despite reduced growth, UV exposure promotes acclimation to environmental stressors, revealing a trade-off between growth and acclimation. These findings challenge previous assumptions regarding UV-B susceptibility in shade-adapted old forest lichens and underscore the intricate interplay between lichen growth and acclimation.

## Introduction

Ultraviolet (UV) radiation is a fundamental component of solar radiation (Gates 1965) influencing the evolution of organisms since the dawn of life (Rothschild 1999). Its influence dates back before the formation of the protective ozone layer (Cockell 1998) and after (Robson et al. 2019). Lichens, renowned for their remarkable UV tolerance, exemplify this evolutionary resilience. Notably, lichens have demonstrated the ability to survive prolonged exposure to UV-C in outer space (Brandt et al. 2015). This exceptional resilience is particularly evident in extremotolerant lichen species, which thrive in some of the harshest environments. Their survival is closely linked to specific morphological traits (Meessen et al. 2013). Among these traits are cortical pigments such as melanin, parietin, and usnic acid, which act as efficient screens against UV-B (Solhaug et al. 2003; McEvoy et al. 2006).

Cortical pigments safeguard lichen photobionts from excess visible light by forming a protective layer on top of the upper cortex (Solhaug and Gauslaa 1996; Gauslaa and Solhaug 2001; Nybakken et al. 2004; McEvoy et al. 2007a; Solhaug et al. 2010) and are strongly induced by UV-B exposure, as reviewed by Solhaug & Gauslaa (2012). While the photobiont demonstrates significant UV-B resilience, the mycobiont may be susceptible to UV-B radiation (Buffoni-Hall et al. 2003), especially given its application in controlling pathogenic fungi on crops (e.g., Suthaparan et al. 2016). Research has largely shown minimal UV-B damage in lichen photobiont (Gauslaa and Solhaug 2004; Solhaug and Gauslaa 2004; Larsson et al. 2009) with adverse effects noted only at unrealistically high UV-B exposures (Gauslaa and Ustvedt 2003; Solhaug et al. 2009; Gauslaa et al. 2017a).

Growth is a robust and ecologically relevant measure of lichen fitness integrating functions of all partners in the symbiotic lichen consortium but has rarely been used to assess effects of UV. UV-B strongly reduced growth rates in two lichens cultivated in growth chambers under artificial light with UV-B supplementation (Chowdhury et al. 2017). No adverse effects were seen on photobiont parameters, suggesting the mycobiont as the more UV-B-sensitive partner. We aim to reassess the impact of UV-A and UV-B on lichen growth rates under field conditions. By using solar filters that selectively transmit either PAR, PAR + UV-A, or PAR + UV-A + UV-B, we seek to overcome the challenges associated with artificial light experiments. Additionally, we will evaluate acclimation traits in the photobiont such as changes in photosynthetic parameters (chlorophylls, quantum yield of CO_2_-uptake (*Φ*_CO2_), maximal yield of photosystem II (*F*_*V*_*/F*_*M*_)), and in the mycobiont (thallus thickness and melanin induction). Our study focuses on sympatric populations of two old forest lichens: the widespread *Lobaria pulmonaria* and the rare, more oceanic *L. virens* (Ellis 2016; Ormond et al. 2025) known for its susceptibility to high light (Gauslaa and Solhaug 1996).

Our objectives are thus threefold: first, to quantify relative mass and thallus area growth rates under three solar radiation regimes in an open sun-exposed environment; second, to assess photobiont viability parameters following 27 days of exposure; and third, to evaluate acclimation responses in the photobiont and mycobiont. We hypothesize a UV-B-driven trade-off in growth and acclimation, positing that successful acclimation to environmental stressors may outweigh the pursuit of maximal growth rates in long-lived lichen species.

## Material and methods

### Lichen material

Two foliose cephalolichens, *Lobaria pulmonaria* (L.) Hoffm. and *L. virens* (With.) J.R. Laundon, also known as *Ricasolia virens* (With.) H.H.Blom & Tønsberg, were collected on June 27, 2015. The samples came from sympatric populations residing on *Quercus* trunks within open, old forests in Langangen, Porsgrunn, Telemark, southern Norway (59°06′43’’N, 9°50′05’’E, 140 m a.s.l.). These forests were abundant with both species. The collected lichens were cleaned from debris, bryophytes, and tree bark. From each species, 82 full-sized lobes—referred to as thalli—were selected. They were air-dried at room temperature and then stored in a freezer at -18°C until experiments commenced on September 01. When collected, the thalli appeared ash-gray with no signs of melanin in the air-dry state, although *L. virens* generally exhibited a slightly darker hue compared to *L. pulmonaria*. Notably, *L. virens* was richly fertile with apothecia present on all thalli, whereas *L. pulmonaria* was sorediate and sparingly fertile.

Prior to and following the field experiment, the air-dry mass of each thallus (± 0.1 mg) was measured after 24 h drying at room temperature. To account for variations due to air humidity, five additional thalli from each species were weighed air-dry during each measuring period. These control thalli were then dried at 70 °C for 24 h and their oven-dry mass (DM) was immediately reassessed. The average change in mass observed in the control thalli during oven drying was used to calculate the DM of all experimental thalli. After weighing, the experimental thalli were sprayed with deionized water, and their surface areas (A) were measured using a Li-Cor leaf area meter (LI3100, Lincoln, NE, USA).

### Growth experiment

**Fig. 1:**
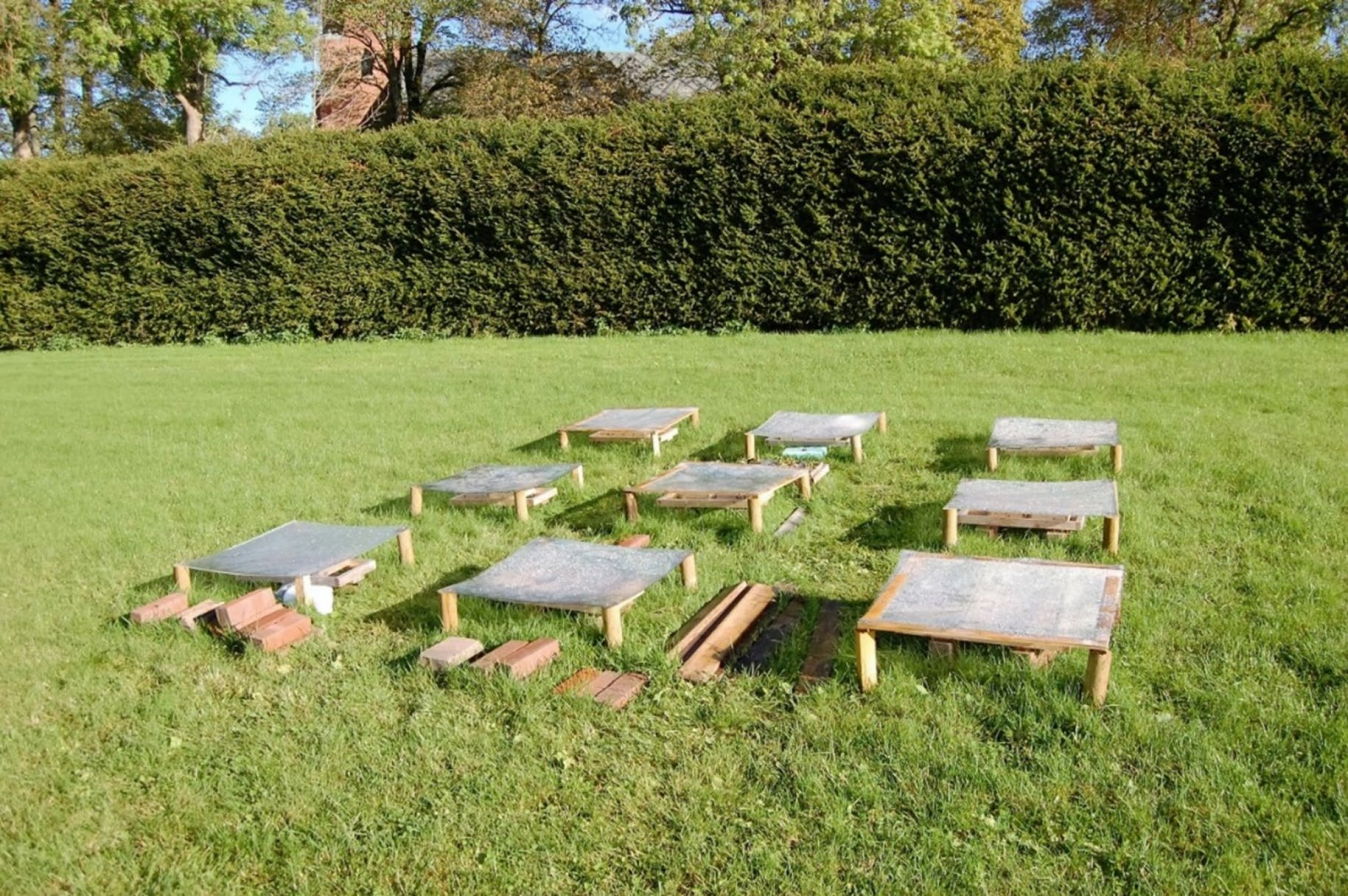
Field setup for the growth experiment: Fifty-four thalli from each lichen species were placed under solar radiation screens—polycarbonate (transmitting PAR only), polyester (transmitting PAR + UV-A), and acryl (transmitting PAR + UV-A + UV-B)—each replicated three times. Thalli were sewn onto plastic grids, mounted on nine 14 × 18 cm wooden frames, with 6 thalli of each species on each frame situated 20 cm above the grass cover

**Fig. 2:**
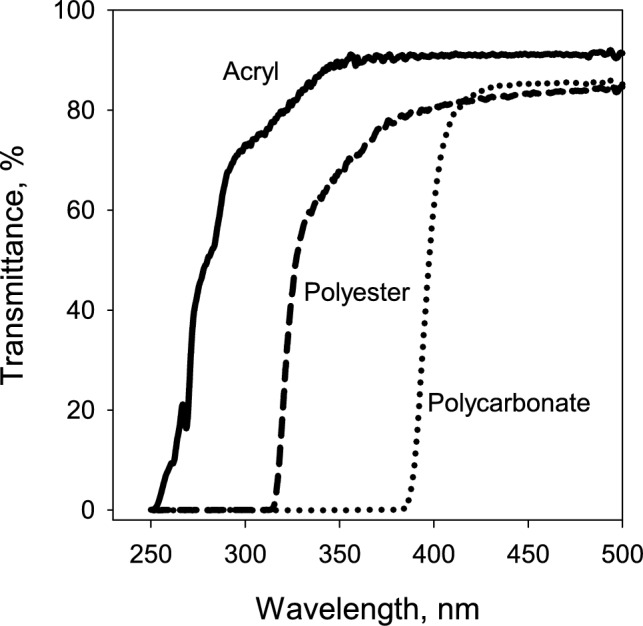
Average transmittance spectra (*n* = 3) for the following solar radiation screens: polycarbonate, polyester, and acryl screens used to alter the spectral composition of natural solar radiation during the field experiment. Each screen type selectively filtered portions of the spectrum to assess the impact of different light regimes on lichen growth

Fifty-four thalli from each species were randomly selected and sewn with a thin thread onto plastic grids, which were mounted on nine 14 × 18 cm wooden frames. Each frame held six thalli of each species. The frames were positioned 20 cm above a grass cover in an open, southwest-facing lawn, situated 30 m south of a trimmed hedge of *Picea abies* in Ås, southeastern Norway (59°40′N, 10°47’E, 100 m a.s.l.). They were placed under three types of screens (Fig. 1), each with three replications: 1) polycarbonate screen that blocked the radiation < 400 nm while transmitting photosynthetically active radiation (PAR) (clear polycarbonate, 3mm, Finn Løken AS, Ås, Norway); 2) polyester screen, blocking the radiation < 315 nm, transmitting PAR + UV-A (PET, 0·175mm, Nordbergs Tekniska AB, Vallentuna, Sweden); and 3) acryl screen, blocking radiation < 250 nm, transmitting PAR + UV-A + UV-B (Acrylic sunbed, 3mm, Finn Løken AS, Ås, Norway). The screens were installed 10 cm above the lichens to allow natural air circulation, preventing excessive heating. The transmittance spectra of these screens are shown in Fig. 2. The experimental setup allowed for similar PAR level across different UV treatments, decoupling the natural correlation between PAR and UV, thus isolating UV-specific effects.

The setup commenced on September 03, 2015, and the lichens were harvested on September 30, 2015. September was chosen as the experimental period due to its optimal conditions for lichen growth, characterized by the following: 1) longer nights that facilitate strong nocturnal cooling, providing lichens with moisture (Gauslaa 2014), 2) sufficiently high temperatures and daylight conducive to robust lichen growth rates (Larsson et al. 2012), 3) a low likelihood of extreme, prolonged sunny, and dry periods that could be detrimental to *Lobaria* species (Gauslaa and Solhaug 1996), 4) fully developed transpiring foliage in surrounding vegetation, enhancing ambient air moisture, and 5) a solar elevation high enough to ensure reasonable UV radiation exposure.

Throughout the experiment, thalli were sprayed with deionized water every morning and evening, except during rainy days. Environmental parameters such as air temperature, relative humidity, and photon flux density (PAR) were recorded at the lichen location beneath an acrylic sunbed, which transmitted PAR and UV. Data were logged every min using a Hobo H21-002 Micro Station Data Logger (Onset Computer Corporation, Bourne, MA, USA). UV radiation (295–385 nm) was recorded every 10 min using an Eppley Ultraviolet radiometer (The Eppley Laboratory, Inc., Newport, Rhode Island USA). UV data and rainfall measurements were collected from Ås meteorological station situated 1.3 km from the study site, operated by the Department of Mathematical Sciences and Technology, NMBU.

**Fig. 3:**
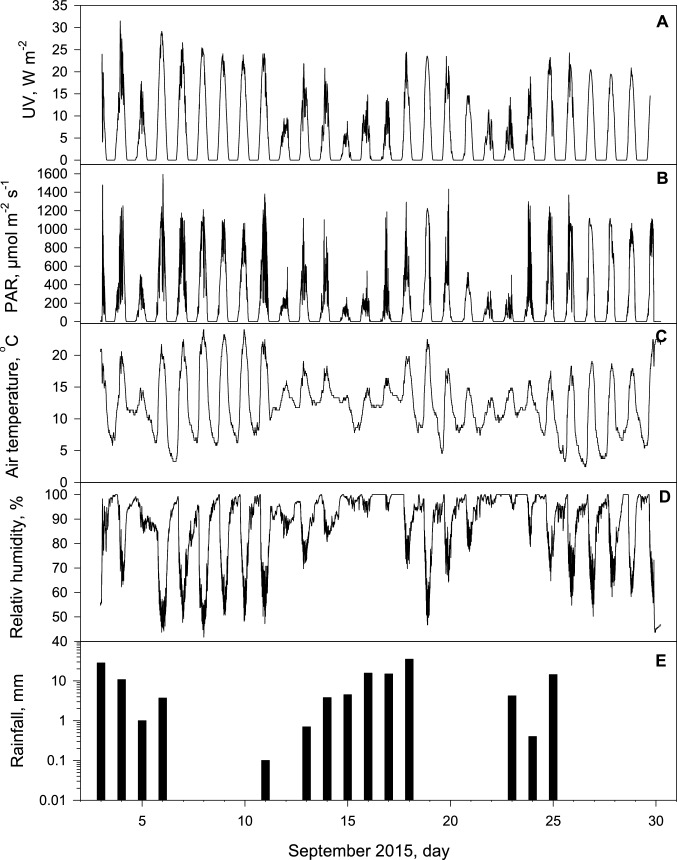
**A** Ultraviolet (UV) radiation, **B** photosynthetic active radiation (PAR), **C** air temperature, **D** relative humidity, and **E** daily rainfall during the 27 days field experiment

During the experiment, most days were partly sunny, with maximum daily PAR levels reaching 1100 µmol photons m^−2^ s^−1^ or higher, peaking at 1593 µmol photons m^−2^ s^−1^ (Fig. 3). Seven days were cloudy, with PAR falling below 600 µmol photons m^−2^ s^−1^, and 14 days experienced rainfall. During daytime, the average PAR was 357 µmol photons m^−2^ s^−1^. Beneath the screens, maximum daily temperatures did not exceed 24 ºC, but strong nocturnal cooling often reduced temperatures to below 8 ºC after sunny days, leading to relative humidity (RH) nearing or reaching 100% most nights (Fig. 3). The air temperature at the lichen level near the ground exceeded 20 ºC on 10 days (Fig. 3), whereas nearby meteorological data at 2 m height showed maximum air temperatures exceeding 20 ºC on only 3 days, never surpassing 20.8 ºC. Extensive dewfall often occurred at the end of clear nights, wetting lichens under the UV screens. The mean UV during daytime was 6.55 W m^−2^ (Fig. 3), with the maximum level recorded being 31.5 W m^−2^.

We measured relative growth rate (RGR) = (ln (DM_end_ / DM_start_)) × 1000 / ∆t, in mg g^−1^ d^−1^, and relative thallus area growth rate (RT_A_GR) = (ln (A_end_ / A_start_)) × 100 / ∆t, in mm^2^ cm^−2^ d^−1^, where ∆t is the number of days between start and end of experiment (27 d). We also recorded specific thallus mass (STM = DM / A), which is correlated with the thickness of lichen thalli (Gauslaa & Coxson 2011). Change in STM was calculated before and after the field experiment as ∆STM = (STM_end_ – STM_start_) × 100 / STM_start_.

### Chlorophyll a fluorescence

Before initiating the growth experiment, all thalli were kept hydrated in low light (~ 10 µmol photons m^−2^ s^−1^) at 15 ºC for 24 h. This preconditioning period allowed them to recover from any potential photoinhibition experienced in their natural habitat prior to collection. Each thallus was then dark acclimated for 15 min before recording the maximal yield of photosystem II (*F*_*V*_*/F*_*M*_) using a PAM-2000 fluorometer (Walz, Effeltrich, Germany). Following the harvest on September 30, 2015, the thalli were rehydrated with deionized water and kept in low light (~ 10 µmol photons m^−2^ s^−1^) at 15 ºC for another 24 h. Then thalli were dark acclimated for 15 min before *F*_*V*_*/F*_*M*_ measurements were taken again.

For light response curves, thalli were again hydrated and placed at 15 °C under 10 µmol photons m^−2^ s^−1^ for 24 h, followed by dark acclimation for 15 min. Measurements were conducted using an IMAGING-PAM fluorometer (MAXI version, Walz, Germany) equipped with a red light (650 nm) LED lamp, using ImagingWin software (Heinz Walz, Effeltrich, Germany). PAR was incrementally increased (0, 1, 21, 56, 111, 186, 281, 336, 398 µmol photons m^−2^ s^−1^) at 40 s intervals. At the end of each light step, the effective quantum yield of PSII was measured with a saturating pulse and calculated as *Φ*_PSII_ = (*F*_*M*_*’*- *F*_*t*_) / *F*_*M*_*’*, where *F*_*t*_ is the fluorescence at time *t* and *F*_*M*_*’* is the maximal fluorescence measured during the saturating pulse. The electron transport rate is calculated as ETR = *Φ*_PSII_ × PAR × 0.5 × Abs. 0.5 assumes equal absorption of photons in PSII and PSI, while Abs represents the fraction of incident light absorbed by both photosystems. We assessed the apparent ETR (ETR_App_), which omits the Abs parameter due to its unknown value in lichens. Therefore, ETR_App_ is higher than the actual ETR, indicating increased screening of PAR by cortical pigments.

### CO_2_ uptake

CO_2_ uptake was assessed in February 2016 using lichens that had been stored at -18 ºC since the conclusion of the experiment. Ten thalli from each species per treatment were evaluated, along with control thalli that had been frozen since their initial collection in the field. Prior to conducting gas exchange measurements, eight thalli (one from each treatment plus one control from each species) were retrieved from the freezer, fully hydrated, and placed in low light (~ 10 µmol photons m^−2^ s^−1^) and 15 ºC for 16–24 h.

Measurements of CO_2_ uptake were conducted using a CIRAS-1 infrared gas analyzer (IRGA) (PP Systems, Hoddesdon, UK). The IRGA settings included a CO_2_ concentration of 400 (± 10) ppm, a temperature of 20 °C, no water removal, and a flow rate of 200 ml min^−1^. A LED lamp (Model SL-3500, Photon System Instruments, Brno, Czech Republic) served as the light source. Each thallus was placed in a cuvette and initially exposed to 100 µmol photons m^−2^ s^−1^ for approximately 5 min to activate photosynthesis. Subsequently, the thalli were subjected to incremental light intensities of 0, 50, 100, 200, 400, and 600 µmol photons m^−2^ s^−1^ with equal levels of red, green, and blue light. CO_2_ uptake was recorded once the IRGA readings stabilized, typically around five minutes after adjustment in light intensity. The quantum yield of CO_2_ uptake (*Φ*_CO2_) was estimated from the light response curves with the software Photosyn Assistant, Ver. 1.1.2 (Dundee Scientific, Dundee, UK).

### Lichen color and spectral reflectance

To document lichen colors before and after the experiment, photographs of 72 thalli of each species were taken while air-dry, as melanin pigmentation is more discernible when dry. Since melanin is challenging to extract from lichens, reflectance spectra were employed to quantify this pigment in *L. pulmonaria* and *L. virens* exposed to the three UV regimes. Before measuring reflectance, the hydrated thalli were flattened and dried under light pressure. Reflectance spectra of the dried thalli were recorded using a spectrometer (model SD2000, Ocean Optics, Eerbeek, The Netherlands) connected to an integrating sphere (ISP-50-REFL Ocean Optics) via a 400-µm-thick fiber. Each thallus was positioned on top of the sphere, and a halogen lamp (DH2000, Ocean Optics) illuminated the thallus through a 600 µm optical fiber connected to the sphere. Reflectance percentage calculations were based on a dark spectrum and a reference spectrum from a white reference tile (WS-2, Ocean Optics).

The browning reflectance index (BRI) for each thallus was calculated using the formula from Chivkunova et al. (2001): BRI = (1 / *R*_*550*_ – 1 / *R*_*700*_) / *R*_*750*_, where *R*_*550*_, *R*_*700*_, and *R*_*750*_ are the reflectance values at 550, 700, and 750 nm, respectively. BRI has been applied to lichens by McEvoy et al. (2007b).

### Chlorophyll measurement

The thalli used for CO_2_ uptake assessments were used for quantifying chlorophylls (Chl). Two discs, each 6 mm in diameter, were cut from each hydrated thallus using a cork borer and placed in an Eppendorf tube containing 1.5 ml of dimethyl sulfoxide. These tubes were subjected to a VWR Ultrasonic cleaner at 60 °C for 30 min. The absorbance of each solution was measured at 649, 665, and 750 nm using a UV 2001 spectrophotometer (Shimadzu, Japan). To correct for impurities, the absorbance at 750 nm was subtracted from the absorbance readings at 649 nm (A_649_) and 665 nm (A_665_). The Chl content of each thallus was calculated using Wellburn (1994) formulas: Chl *a* = 12.19 * A_665_ ˗ 3.45*A_649_ and Chl *b* = 21.99 * A_649_ ˗ 5.32*A_665_. Chl *a/b*-ratios were calculated on a mass basis.

### Statistical analyses

After the experiment, one *L. pulmonaria* thallus (PAR + UV-A + UV-B) and two *L. virens* thalli (PAR only) were identified as outliers due to unrealistically high increases in STM, likely caused by errors in area quantification. These outliers also exhibited abnormal reductions in *F*_*V*_*/F*_*M*_ during the experiment and were excluded from further analyses.

Statistical analyses were run using Jamovi 2.3.28. Initial analyses involved linear mixed effect models for the measured variables, treating screen replication as a random factor. However, this factor was not significant, as indicated by minor differences between *R*^2^_marginal_ and *R*^2^_conditional_ values. Consequently, subsequent analyses excluded the random factor, employing generalized linear models (GLMs) with species and treatments as factors, including the interaction term when significant. Variables were log transformed as needed.

One-way ANOVAs were utilized to analyze differences between treatment groups for the parameters such as RGR, RT_A_GR, ∆STM, BRI, *Φ*_CO2_, total Chl content, and the Chl *a/b*-ratio. BRI data were log_10_ transformed to meet ANOVA requirements, and Tukey’s method was employed for mean comparisons between groups. A paired *t*-test analyzed changes in *F*_*V*_*/F*_*M*_ before and after the growth experiment, while a two-sample *t*-test assessed contrasts in Chl *a* + *b*, *Φ*_PSII_, STM_start_, RGR, RT_A_GR, and ∆STM between the two species. The significance level for all tests was set to 0.05. Mean ± standard error (SE) is given in text, figures, and tables.

## Results

### UV-induced changes in lichen color

**Fig. 4:**
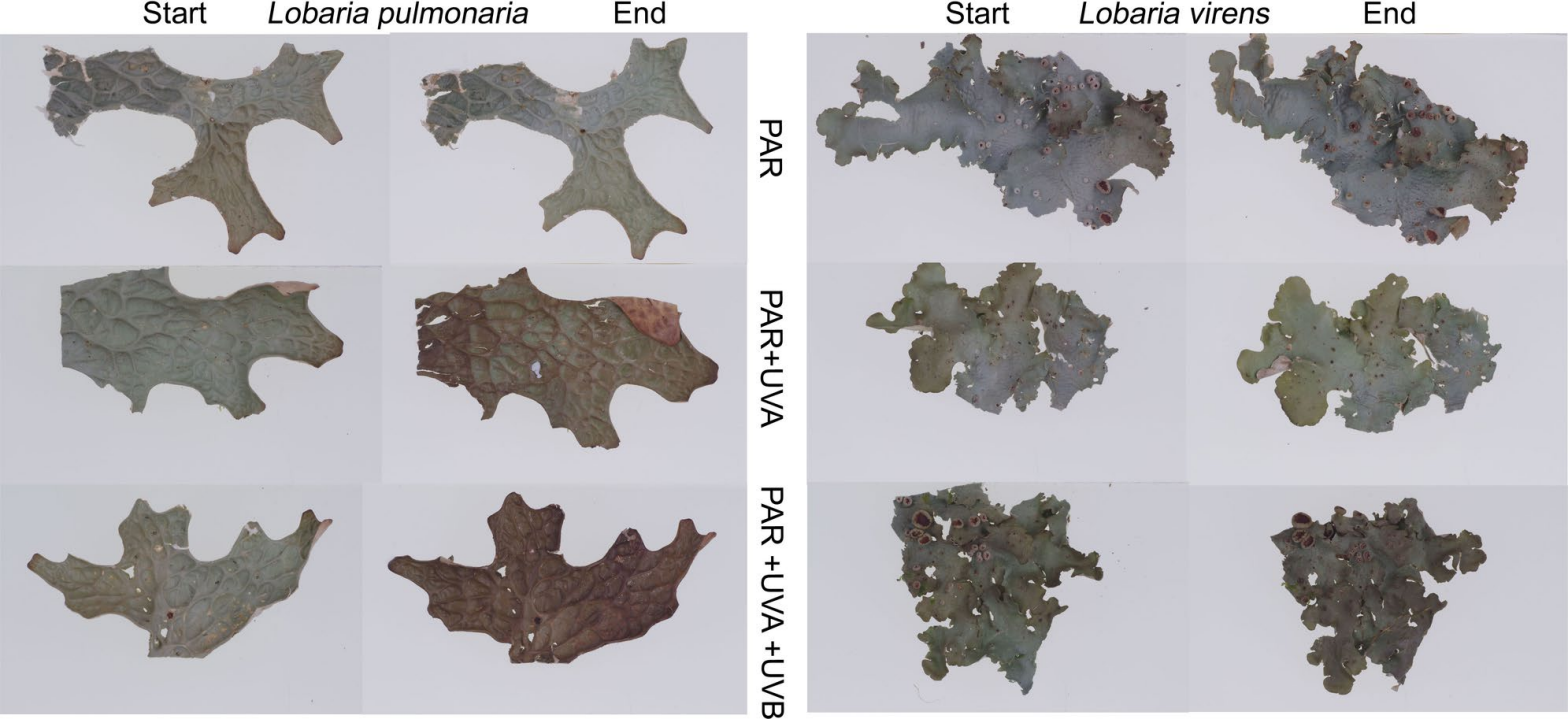
Photographs of lichen thalli before and after exposure to different radiation treatments in the field: 1) PAR (upper row), PAR + UV-A (middle row), and PAR + UV-A + UV-B (lower row). The left side displays *Lobaria pulmonaria*, while the right side shows *Lobaria virens*. These images highlight the typical color variations induced by each light regime

**Fig. 5:**
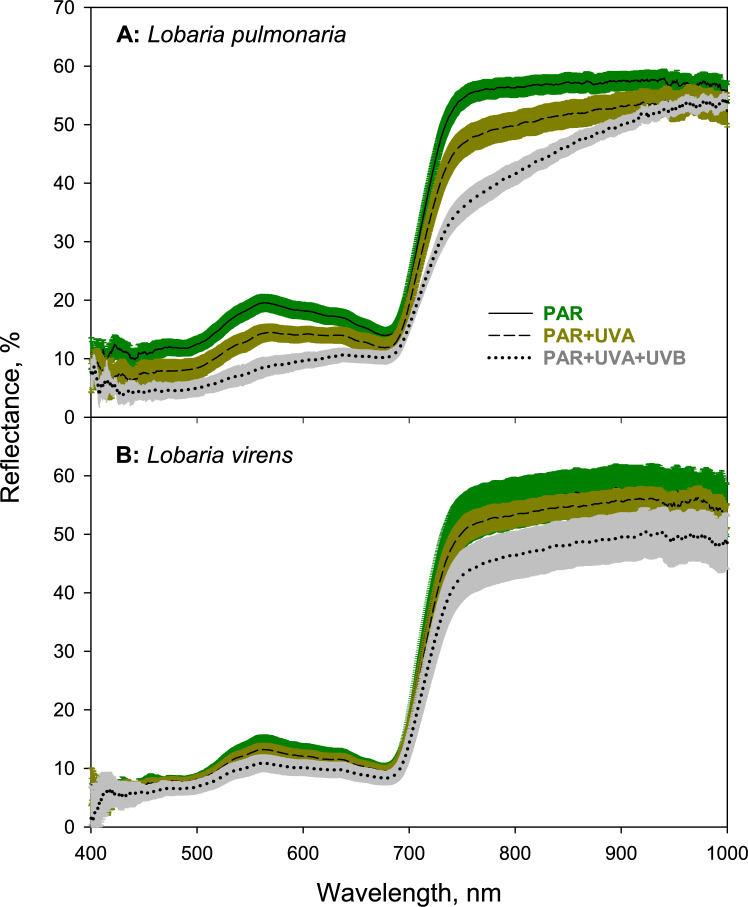
Visible and near infrared reflectance spectra (400–1000 nm) for **A** *Lobaria pulmonaria* and **B** *Lobaria virens* following 27 days of exposure to three solar radiation treatments in an open habitat: PAR (uppermost graph), PAR + UV-A (middle graph), and PAR + UV-A + UV-B (lower graph)

Natural UV radiation at the open transplantation site altered lichen colors in wavelength- and species-specific ways (Fig. 4 and 5). Lichens shielded from UV retained their pale coloration despite higher PAR exposure compared to their original forested habitat. Within-species color changes were significant, as demonstrated by non-overlapping 95% confidence intervals of radiation treatment-specific reflectance spectra in parts of the visible and near infrared spectrum (Fig. 5). UV exposure led to the disappearance of the green reflectance peak at 560 nm in *L. pulmonaria* and its weakening in *L. virens,* along with a reduction in the reflectance minimum at approximately 680 nm, and finally a reduced reflectance in the short-wave infrared spectrum (Fig. 5). The color change was more pronounced in *L. pulmonaria* manifested as UV-induced browning, which was noticeable with UV-A treatment and intensified with UV-B exposure. By contrast, *Lobaria virens* showed browning only under full sun spectrum (PAR + UV-A + UV-B) (Fig. 4). The reflectance was lower in *L. virens* (Fig. 5), evidenced by its slightly darker gray appearance (Fig. 4).

**Fig. 6:**
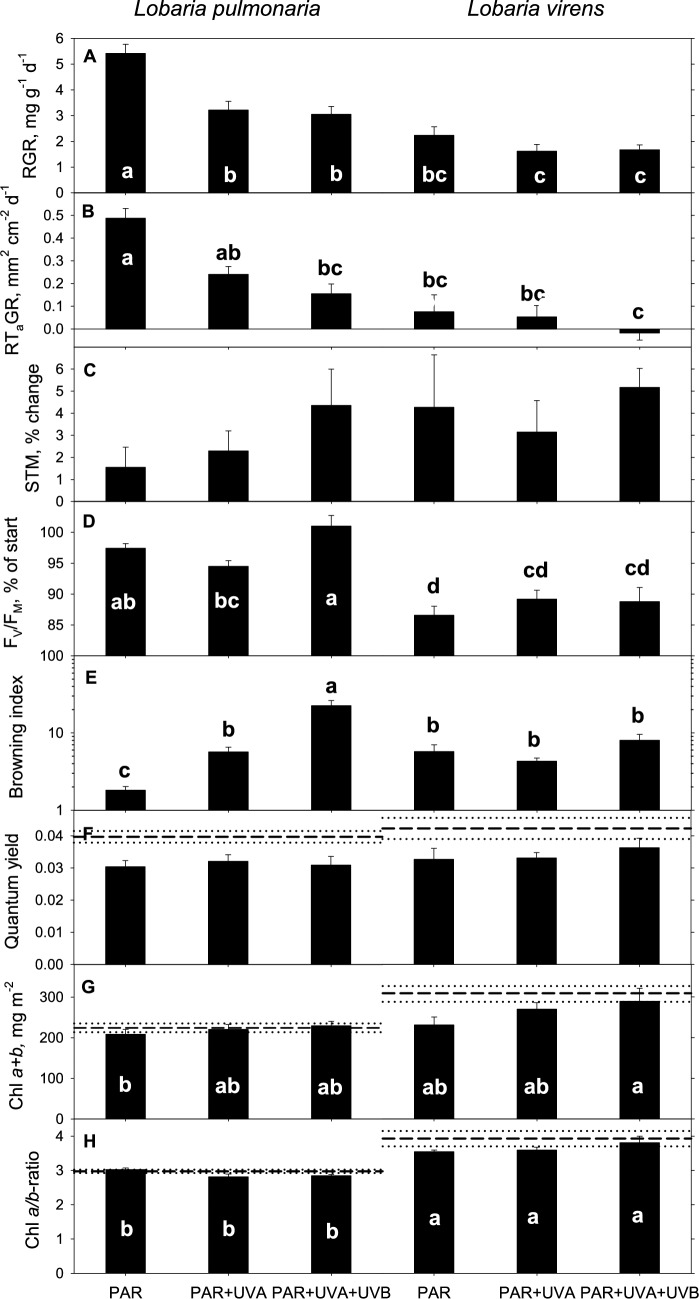
Measured responses of *Lobaria pulmonaria* (left) and *Lobaria virens* (right) exposed to three solar radiation treatments (PAR, PAR + UV-A, and PAR + UV-A + UV-B, as indicated on the x-axis) over 27 days in an open field position. The responses measured include **A** Relative growth rate (RGR), **B** Relative thallus area growth rate (RT_A_GR), **C** percent change in specific thallus mass (STM), **D** maximal yield of photosystem II (*F*_*V*_*/F*_*M*_) expressed as a percentage of initial values, **E** Browning index (BRI), **F** quantum yield of CO_2_ uptake (*Φ*_CO2_), **G** Total chlorophyll content per area (Chl *a* + *b*), and **H** Chl *a/b*-ratio. Columns represent mean values with error bars indicating standard errors. Horizontal hatches lines in panels **F–H** denote mean initial values from control thalli originating from the forested source habitat with dotted lines showing ± 1 standard error. Means that differ significantly (*P* < 0.05) are marked with different letters

The browning index (BRI) varied significantly across radiation treatments for *L. pulmonaria*, but not for *L. virens* (Fig. 6E). In *L. pulmonaria*, BRI increased with PAR + UV-A treatment, with UV-B exerting an additional strong effect. Due to the exponential increase in BRI with UV exposure, BRI was log transformed in Fig. 6E and statistical analyses (Table 1). *Lobaria virens* exhibited similar BRI across all treatments, matching the BRI of *L. pulmonaria* exposed to PAR + UV-A (Fig. 6).

**Table 1:** Summary statistics of the two cephalolichens *Lobaria pulmonaria* and *L. virens*

	*Lobaria pulmonaria*			*Lobaria virens*			GLM; *P*-level			
	Mean ± 1SE	Min	Max	Mean ± 1SE	Min	Max	Species	Treatment	S × T	*R*^2^_adj_
Relative growth rate (RGR), mg g^−1^ d^−1^	3.91 ± 0.24	1.10	7.80	1.84 ± 0.16	− 1.18	4.1	< 0.001	< 0.001	0.007	0.499
Relative area growth rate (RT_A_GR), mm^2^ cm^−2^ d^−1^	0.297 ± 0.030	− 0.07	1.01	0.036 ± 0.031	− 0.506	0.520	< 0.001	0.0016	0.022	0.417
Specific thallus mass at start (STM_start_). mg cm^−2^	12.7 ± 0.5	5.5	31.8	15.4 ± 0.4	9.8	23.9	< 0.001	0.692	0.820	0.133
STM, % change	2.70 ± 0.69	− 7.8	19.7	4.19 ± 0.92	− 10.8	22.1	0.206	0.281	0.747	0.000
*F*_*V*_*/F*_*M*start_	0.701 ± 0.004	0.558	0.733	0.664 ± 0.004	0.557	0.714	< 0.001	0.411	0.268	0.421
*F*_*V*_*/F*_*M*end_, % of start	97.6 ± 0.76	85.4	125.6	88.3 ± 1.0	64.4	108.8	< 0.001	0.078	0.055	0.379
Browning index (BRI)	9.8 ± 1.7	0.6	64.5	6.0 ± 0.7	1.3	23.9	0.330	< 0.001	< 0.001	0.568
Thallus area (A_start_), cm^−2^	10.5 ± 0.4	5.2	16.8	6.3 ± 0.3	3.1	13.1	< 0.001	0.108	0.621	0.436
Dry mass (DM_start_), mg	130.4 ± 5.3	65.7	230.1	98.0 ± 5.8	31.6	207.4	< 0.001	0.360	0.672	0.142
Chlorophyll *a* + *b*, mg m^−2^	219 ± 7	143	295	264 ± 14	155	490	0.009	0.078	0.688	0.125
Chlorophyll *a/b*-ratio	2.89 ± 0.04	2.3	3.0	3.64 ± 0.07	3.0	4.7	< 0.001	0.337	0.100	0.644
Quantum yield of CO_2_ uptake (*Φ*_CO2_)	0.031 ± 0.001	0.018	0.050	0.034 ± 0.002	0.19	0.58	0.190	0.679	0.655	0.000

*N* = 54 for RGR, RT_A_GR, STM_start_, STM (% change), F_V_/F_Mstart_, F_V_/F_Mend_ (% of start), BRI, A_start_, and DM_start_. *N* = 30 for Chlorophyll *a* + *b* and Chl *a/b*-ratio. Parameters with positive values were Box-Cox transformed in the GLM

### UV radiation effects on lichen growth rates

UV radiation significantly influenced lichen growth rates, with responses varying between species (Fig. 6). Initial differences in thallus size (DM and A), thickness (STM), and *F*_*V*_*/F*_*M*_ between the species were significant (Table 1; *P* < 0.001) due to their different morphologies. Thalli selected for the three UV treatments showed no significant initial variation (*P* > 0.1), optimizing the chance to detect treatment effects.

The mean relative growth rate of *L. pulmonaria* (RGR = 3.91 ± 0.24 mg g^−1^ d^−1^; mean ± SE; *n* = 54) was 2.1 times higher (*P* < 0.001) than that of *L. virens* (1.84 ± 0.16 mg g^−1^ d^−1^; Table 1). Similarly, the mean relative thallus area growth rate (RT_A_GR) of *L. pulmonaria* (0.297 ± 0.030 mm^2^ cm^−2^ d^−1^) was 8.3 times higher (*P* < 0.001) than that of *L. virens* (0.036 ± 0.031 mm^2^ cm^−2^ d^−1^; Table 1). Over 27 days, *L. pulmonaria* increased by 11.3 ± 0.7% in mass (total range 3.0 – 23.4%), while *L. virens* grew by 5.2 ± 0.4% (-3.1 – 11.6%). UV radiation reduced lichen growth rates in both species, with the greatest impact on the faster-growing *L. pulmonaria* (Fig. 6A–B; Table 1). The reduction in RGR was similarly strong under UV-A and UV-A + UV-B exposure, but RT_A_GR appeared more severely affected by UV-A + UV-B than by UV-A alone (Fig. 6A–B). In *L. pulmonaria*, RGR during the UV-A + UV-B exposure fell to 56% compared to PAR alone, while RT_A_GR declined to 31%.

**Fig. 7:**
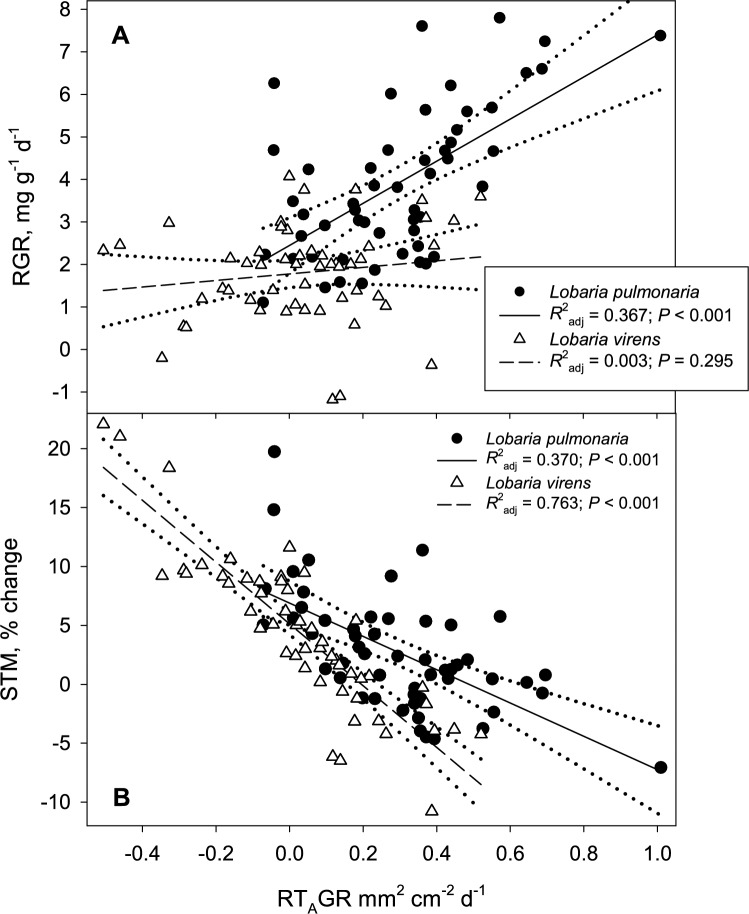
The relationships between **A** relative growth rate (RGR) and **B** change in specific thallus mass (STM) versus relative thallus area growth rate (RT_A_GR) in *Lobaria pulmonaria* (filled circles and solid line) and *Lobaria virens* (open triangles and hatched line) after a 27 day growth period in an open, sun-exposed environment. Dotted lines indicate the 95% confidence intervals

The relationship between growth in area and mass differed markedly between the two species. *Lobaria pulmonaria* displayed a strong positive correlation between RGR and RT_A_GR (Fig. 7A). In contrast, *L. virens* showed a decoupled relationship between area and biomass changes (Fig. 7A). Instead, the change in thallus thickness (% change in STM) decreased more sharply with increased RT_A_GR in *L. virens* compared to *L. pulmonaria*, as indicated by a steeper slope in its regression line and a higher *R*^2^_adj_ (Fig. 7B).

Initially, thallus thickness (STM_start_) was significantly greater in *L. virens* (15.3 ± 0.4 mg cm^−2^) compared to *L. pulmonaria* (12.7 ± 0.5 mg cm^−2^; *P* < 0.001; Table 1). Both species experienced significant increases in STM across all treatments during the experiment, but the increase did not significantly differ between them (∆STM = 4.19 ± 0.92% in *L. virens*; 2.70 ± 0.69% in *L. pulmonaria*; *P* = 0.206). Although the largest increases in STM occurred during the UV-B treatment (Fig. 7C), radiation treatments did not yield statistically significant effects (*P* = 0.281; Table 1).

### UV radiation and lichen chlorophyll

*Lobaria virens* had higher Chl content compared to *L. pulmonaria* (*P* = 0.009; Table 1). During the growth study, there was a noticeable reduction in both total Chl (Fig. 6G) and the Chl *a/b*-ratio (Fig. 6 H) in PAR-treated *L. virens* thalli, while UV-B-treated thalli did not show this reduction. This is evident from the gap between the hatched lines (representing mean start values) and the columns (representing mean end values). In contrast, total Chl content and Chl *a/b*-ratio in *L. pulmonaria* remained stable during the experiment (Fig. 6G–H). There was a weak (*P* = 0.037; Table 1) overall increase in total Chl with increasing UV exposure (Fig. 6G), while the Chl *a/b*-ratio remained constant across radiation treatments (Table 1; Fig. 6H). The thicker *L. virens* showed a marginally higher Chl *a/b*-ratio than the thinner *L. pulmonaria* (*P* < 0.001; Table 1). These findings suggest that UV-A and UV-B exposure did not have adverse effects on chlorophyll.

### Induction of melanic pigments reduced photoinhibition and growth

**Fig. 8:**
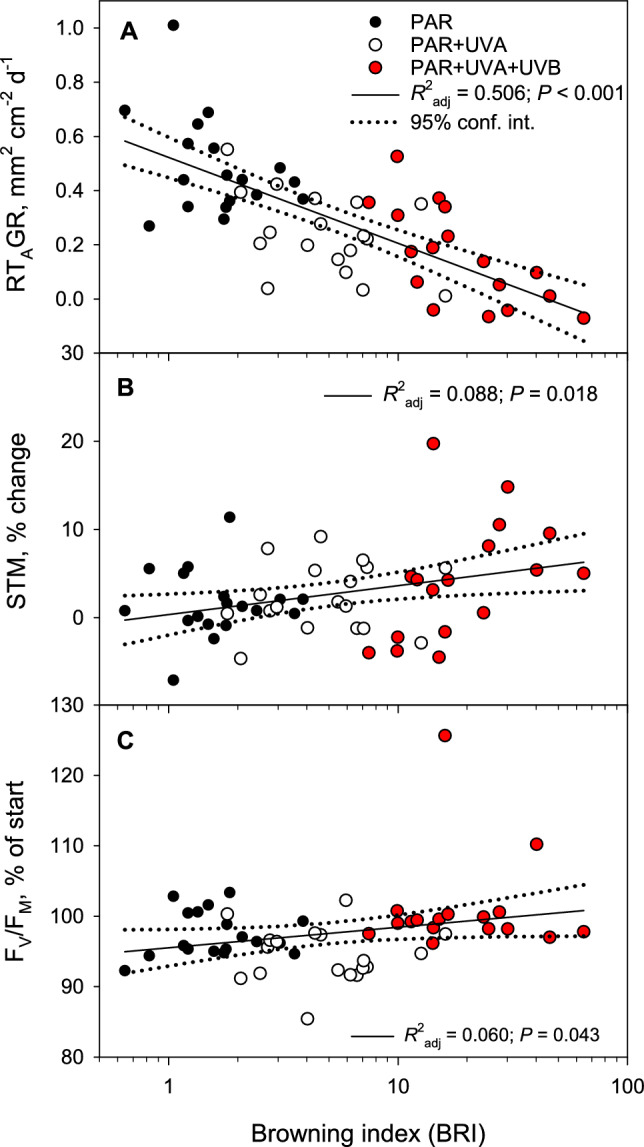
The relationships between **A** relative thallus area growth rate (RT_A_GR), **B** change in specific thallus mass (STM), and **C** maximal yield of photosystem II (*F*_*V*_*/F*_*M*_) expressed as percentage of initial values versus melanic pigmentation measured by the browning index (BRI) in shade-acclimated thalli of *Lobaria pulmonaria* after 27 days of growth in an open, sun-exposed environment. Dotted lines represent the 95% confidence intervals for these relationships. Symbols are color coded according to the solar radiation treatments: PAR (black), PAR + UV-A (white), and PAR + UV-A + UV-B (red)

Before the growth experiment, *L. virens* exhibited slightly stronger photoinhibition than *L. pulmonaria* as indicated by its lower initial *F*_*V*_*/F*_*M*_ (0.664 ± 0.004 versus 0.701 ± 0.004; Table 1). Despite experiencing less browning than *L. pulmonaria*, *L. virens* faced higher photoinhibition during the growth experiment, achieving only 88.3 ± 1.0% of its initial *F*_*V*_*/F*_*M*_ levels. Conversely, *Lobaria pulmonaria*, which demonstrated a robust and dynamic melanin synthesis, effectively mitigated further photoinhibition as evidenced by its significantly higher *F*_*V*_*/F*_*M*_ at the end of the experiment (97.6 ± 0.8% of initial values; Fig. 6D). Radiation treatments did not significantly affect photoinhibition levels in either species (*P* = 0.078; Table 1). By the experiment’s conclusion, the mean *F*_*V*_*/F*_*M*_ was highest in *L. pulmonaria* exposed to both UV-B and UV-A, reaching 101% relative to initial values (Fig. 6D). UV-B radiation did not cause additional photoinhibition compared to PAR alone in either species (Fig. 6D). In *L. pulmonaria*, *F*_*V*_*/F*_*M*_ showed a weak positive correlation with the BRI by the end of the experiment (*R*^2^_adj_ = 0.060; *P* = 0.043; Fig. 8C), suggesting that increased browning contributed to reduced photoinhibition. However, this correlation was not observed in *L. virens*, which experienced less browning.

In *L. pulmonaria*, the expansion of thallus area (RT_A_GR) significantly decreased as the BRI increased (*R*^2^_adj_ = 0.506; *P* < 0.001; Fig. 8A). Similarly, the RGR also declined with BRI, though less strongly (*R*^2^_adj_ = 0.310; *P* < 0.001). Conversely, the change in thallus thickness showed a positive correlation with BRI (*R*^2^_adj_ = 0.088; *P* = 0.018; Fig. 8B). In contrast, *L. virens*, which exhibited less dynamic melanin formation, showed no significant changes in growth with varying BRI (data not shown). This suggests that the relationship between melanin induction and growth is more pronounced in *L. pulmonaria* than in *L. virens*.

### Light response curves of CO_2_ uptake and apparent electron transport rate

**Fig. 9:**
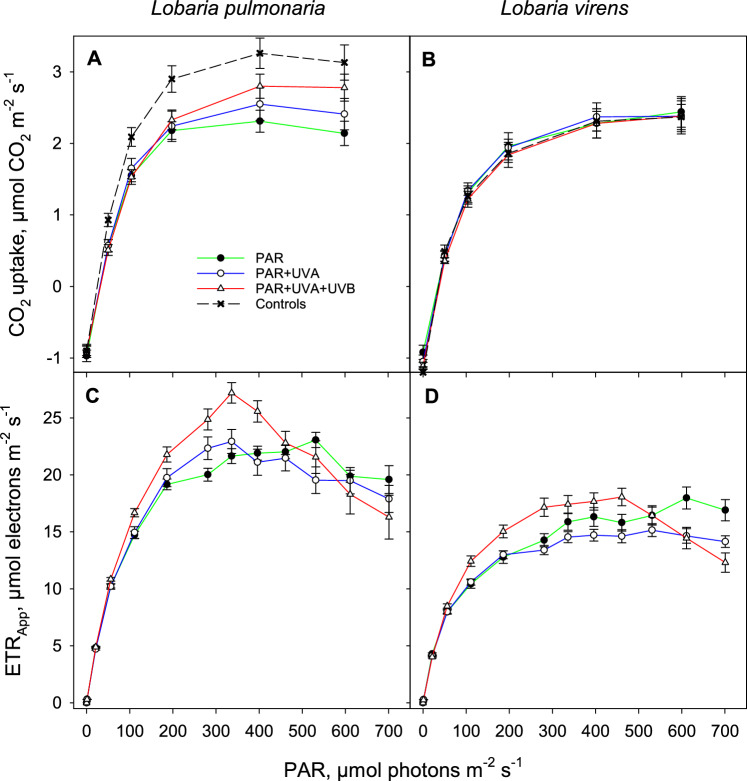
The light response curves for CO_2_ uptake (**A-B**) and apparent electron transport rate (**C-D**) in *Lobaria pulmonaria* (**A, C**) and *Lobaria virens* (**B, D**). These measurements were taken after the shade-acclimated thalli had grown for 27 days in an open, sunny site under three solar radiation treatments: PAR (filled circles, green line), PAR + UV-A (open circles, blue line), and PAR + UV-A + UV-B (open triangles, red line). The hatched black line represents CO_2_ uptake measured prior to transplantation. Vertical bars indicate standard errors; *n* = 10)

In both species, the quantum yield of the CO_2_ uptake (*Φ*_CO2_) was significantly higher in the control thali from the forest than for those that had been exposed in the field experiment. However, there were no differences across the radiation regimes or between the species (Fig. 6F). The highest CO_2_ uptake rates, 3.3 and 3.1 µmol CO_2_ m^−2^ s^−1^, were observed in *L. pulmonaria* controls from the forested habitat at 400 and 700 µmol photons m^−2^ s^−1^, respectively (Fig. 9A). After the growth experiment, these rates slightly decreased in *L. pulmonaria*, while *L. virens* maintained stable uptake rates (Fig. 9A-B). Averaged across treatments, the CO_2_ uptake rates at each PAR level used in the light response curve (Fig. 9A-B) did not differ between the species.

The light response curves in *L. virens* remained consistent between treatments and controls, achieving light saturation at 400 µmol photons m^−2^ s^−1^ (Fig. 9B). Conversely, radiation treatments impacted the CO_2_ uptake of *L. pulmonaria* at higher PAR (400 and 600 µmol photons m^−2^ s^−1^; Fig. 9A). *Lobaria pulmonaria* exposed only to PAR, and thus without melanin formation during the experiment (Fig. 4), reached light saturation at just 200 µmol photons m^−2^ s^−1^. In contrast, individuals exposed to UV-A or UV-A + UV-B, which promoted melanin synthesis, required 400 µmol photons m^−2^ s^−1^ for saturation. The highest CO_2_ uptake rates in light-saturated *L. pulmonaria* thalli, measuring 2.8 µmol CO_2_ m^−2^ s^−1^ at 400 and 700 µmol photons m^−2^ s^−1^, were recorded following the PAR + UV-A + UV-B treatment (Fig. 9A).

ETR_App_ was significantly higher in *L. pulmonaria* (Fig. 9C) compared to *L. virens* (Fig. 9D). Within each species, thalli exposed to PAR + UV-A + UV-B, which prompted the strongest melanin synthesis (Fig. 4), exhibited the highest ETR_App_. At PAR levels exceeding light saturation, both species experienced the most pronounced reduction in ETR_App_ in thalli treated with PAR + UV-A + UV (Fig. 9C-D). This decline was notably strong in *L. pulmonaria*, which had thinner thalli (lower STM_start_; Table 1) compared to *L. virens*.

## Discussion

### Lichen growth rates

While growth rates for *L. virens* have not been previously documented, *L. pulmonaria* is recognized for its fast growth compared to members of the Lecanorales (Gauslaa and Goward 2012; Bidussi et al. 2013b; Chowdhury et al. 2017) and Teloschistales (Larsson et al. 2009; Gauslaa et al. 2021). Our field-based RGRs for *L. pulmonaria*, even under the PAR + UV-A + UV-B regime, exceed those reported in natural habitats in e.g., British Columbia (RGR = 0.7–1.3 mg g^−1^ d^−1^; Gauslaa and Goward 2012; Gauslaa and Goward 2020; Bidussi and Gauslaa 2015; Chowdhury et al. 2017) and Spain (0.5–1.3 mg g^−1^ d^−1^; Merinero et al. 2015). They also surpass rates achieved under good growth conditions in Norway (1.5–2.4 mg g^−1^ d^−1^; Larsson et al. 2012). Our rates are comparable to those observed for *L. pulmonaria* in growth cabinet experiments (RGR = 4–11 mg g^−1^ d^−1^; Bidussi et al. 2013a; Gauslaa et al. 2021), though slightly lower than those achieved in cabinets with added UV-B (4.5 mg g^−1^ d^−1^; Chowdhury et al. 2017) or mineral nutrients (10 mg g^−1^ d^−1^; Gauslaa et al. 2021), and in winter-acclimated thalli grown at 12 °C in the lab (7.2 mg g^−1^ d^−1^; Larsson et al. 2009).

The elevated growth rates in our field experiment can be attributed to the favorable seasonal conditions in September, including strong nocturnal cooling and dewfall, which enhanced moisture availability (Gauslaa 2014), and with no excessive heating (Gauslaa and Solhaug 1999). The open site allowed greater nocturnal cooling compared to forest environments. High nocturnal hydration during clear nights contributed to the high growth rates reported in transplanted *L. pulmonaria* on artificial stands in open clear cuts (Gauslaa et al. 2006; 2007). Nighttime hydration boosts lichen growth (Bidussi et al. 2013a) as moist predawn periods at low light optimize relaxation of photoinhibition following sunny days (Solhaug 2018), enabling maximal photosynthesis in early daylight hours before desiccation impacts metabolism. We observed significant dewfall trapped under the screens above the transpiring grass cover in the morning. Even during rainy weather, lichen photosynthesis was hardly limited by low light in the open experimental site, unlike in forests. The screens also shielded lichens from the rain, preventing supersaturation depression (Lange et al. 2001).

In general, the effects of UV on growth are stronger in organisms grown under controlled conditions compared to those in field settings, or when strong UV supplements are applied (e.g., Searles et al. 2001; Pescheck and Bilger 2020). Nevertheless, the observed reduction in growth due to UV exposure in *L. pulmonaria* aligns with earlier findings from growth cabinet studies (Chowdhury et al. 2017). This reduction also parallels similar declines in growth rates seen in vascular plants exposed to UV-B (Poulson et al. 2006).

### Acclimation

Transferring lichens from shaded forest trunks to sunnier environments necessitates acclimation, particularly in *Lobaria* species known for their high-light susceptibility (Gauslaa and Solhaug 1996). A notable example is *L. pulmonaria*, which rapidly synthesizes melanin, creating a strong PAR screen (Gauslaa and Solhaug 2001) that enhances light saturation in melanic thalli compared to pale ones (Fig. 9A). This melanin, identified as N-rich DOPA melanin (Matee et al. 2016; Mafole et al. 2019a), binds with fungal cell wall components and fortifies the lichen cortex (Daminova et al. 2023).

Higher ETR_App_ in *L. pulmonaria* compared to *L. virens* suggest more efficient solar radiation screening, especially in thalli exposed to PAR + UV-A + UV-B (Fig. 9). Interestingly, no significant differences in *Φ*_CO2_ were observed between treatments (Table 1). While higher actinic light screening by melanin might be expected to reduce *Φ*_CO2_, the higher *F*_*V*_*/F*_*M*_ values observed in thalli exposed to UV-B (PAR + UV-A + UV-B) (Fig. 6) suggest that melanin plays a protective role against photoinhibition. This reduced photoinhibition likely compensates for any potential negative effect of actinic light screening, explaining the stable *Φ*_CO2_ across treatments.

UV-B radiation plays a critical role in inducing melanin synthesis, offering substantial protection against high-light damage (Solhaug et al. 2003), most notably at light levels that saturate photosynthesis. Melanin efficiently shields photobiont cells from damage, provided that its darkened effect does not cause excessive warming from solar radiation (Gauslaa and Solhaug 1999). UV-B’s role in forming effective sunscreens suggests it serves more as a regulatory factor than a stressor (Robson et al. 2015; Hideg et al. 2013). In extreme environments, melanin acts as a beneficial sunscreen, aiding lichen survival (Gauslaa and Goward 2023). However, the high solar energy absorbance of melanin can lead to overheating in warmer climates (Gauslaa 1984; McEvoy et al. 2007a). Although the UV-protective role of melanin has been questioned because the pale, melanin-deficient cortices in shade-acclimated *L. pulmonaria* did not transmit wavelengths below 330 nm, its primary function appears to be the protection against PAR and UV-A (Gauslaa et al. 2017a). Additionally, structural cell wall compounds may contribute to UV protection by binding other UV-absorbing substances (e.g., Mahmud et al. 2024).

In *L. pulmonaria*, rapid UV-B-induced melanin synthesis minimizes photobiont damage, as evidenced by full recovery of *F*_*V*_*/F*_*M*_ and stable Chl content. Mafole et al. (2017) confirmed that melanic thalli of this species avoid photobiont damage. Consistently, across various species, melanic thalli more efficiently avoid photoinhibition compared to pale ones (Mafole et al. 2019b). In contrast, the photobiont in *L. virens*, which exhibited weak melanin formation, experienced significant photoinhibition and reduced Chl content post-exposure, consistent with its greater susceptibility to high light (Gauslaa and Solhaug 1996).

Increased thallus thickness, measured as STM, represents another acclimation strategy. The thickening was most pronounced in *L. virens*, which initially had a higher STM than *L. pulmonaria*. Like sun-acclimated plants that develop thicker leaves (Terashima et al. 2005) and compact plant morphology due to UV-B exposures (Jansen 2002), sun-acclimated lichens show increased STM (Snelgar and Green 1981; Gauslaa and Coxson 2011). Increased STM is often reported in forest lichens transplanted to open habitats (Gauslaa et al. 2006; 2009). Lichen STM shapes the water holding capacity (Gauslaa 2014), impacting the duration of photosynthetic active periods in poikilohydric organisms (Gauslaa et al. 2017b; Phinney et al. 2022). However, high STM reduces water uptake rates, affecting photosynthetic activation in humid air (Phinney et al. 2018; Ås Hovind et al. 2020). *Lobaria virens*, with its higher STM, has a greater internal water holding capacity than *L. pulmonaria* (Longinotti et al. 2017). The thinner *L. pulmonaria*, with lobes elevated above the bark, is more exposed to ambient air humidity, whereas the prostrate and thicker *L. virens* benefits from direct stemflow water exposure, as discussed by Longinotti et al. (2017). The significant association between browning and increased thallus thickness for *L. pulmonaria* suggests that UV-B not only aids light acclimation but also enhances water storage capacity. Similarly, UV-B improves drought tolerance of plants albeit through different mechanisms (Poulson et al. 2002).

### Trade-off between growth and acclimation

UV exposure significantly reduced growth rates, but did not impact photosynthesis or other photobiont processes during the field exposure, aligning with findings by Mafole et al. (2017). However, UV-B can directly damage cellular processes in lichen mycobionts (Buffoni-Hall et al. 2003), necessitating the allocation of resources for repair. Additionally, the metabolic demands associated with melanin synthesis and the allocation of resources to increase STM may incur substantial energetic costs. These processes also involve high N requirements due to the synthesis of N-rich melanin. A documented trade-off between STM and RGR suggests that such expenses can offset growth investments (Gauslaa and Goward 2012). BRI, as an indicator of melanin investments, may help explain reduced growth rates alongside increased pigment synthesis.

A notable distinction between the species is that *L. virens*, which grows more slowly, is less prone to grazing than the faster-growing *L. pulmonaria*, even in areas with abundant lichen-feeding gastropods (Asplund et al. 2010). *Lobaria virens* lacks carbon-based secondary compounds, unlike *L. pulmonaria* (Jørgensen and Tønsberg 2007), but may produce toxic microcystins through its secondary *Nostoc* photobiont, as observed in other Peltigerales members (Kaasalainen et al. 2009; 2012). This investment in costly N-based defense compounds could contribute to the lower growth rate of *L. virens* compared to *L. pulmonaria*.

## Conclusions

UV radiation had beneficial effects on the lichen photobiont, despite notably reducing lichen growth rates. While it might seem reasonable to conclude that the mycobiont is the UV-B-susceptible partner in lichen symbiosis, as suggested by Chowdhury et al. (2017), this assumption may not hold true. Acclimation to stressful environments inherently incurs metabolic costs that favor survival over growth, as noted by Colesie et al. (2014). The UV-B-induced acclimation traits that enable lichens to withstand high light stress and increased evaporative demands also carry metabolic expenses. These costs likely contribute to the observed trade-off between growth rate and the synthesis of melanic pigments and thallus thickening, highlighting the intricate balance between lichen growth and acclimation.

## Data Availability

Data will be available on request.
